# Cytokines unveiled: their impact on oral and multisystem features of lupus erythematosus

**DOI:** 10.3389/fimmu.2025.1585280

**Published:** 2025-07-28

**Authors:** Noha M. Elemam, Iman M. Talaat, Omar A. El Meligy

**Affiliations:** ^1^ Clinical Sciences Department, College of Medicine, University of Sharjah, Sharjah, United Arab Emirates; ^2^ Research Institute of Medical & Health Sciences, University of Sharjah, Sharjah, United Arab Emirates; ^3^ Pathology Department, Faculty of Medicine, Alexandria University, Alexandria, Egypt; ^4^ Pediatric Dentistry and Dental Public Health Department, Faculty of Dentistry, Alexandria University, Alexandria, Egypt

**Keywords:** SLE, cytokines, systemic features, oral features, therapeutic targets

## Abstract

Systemic Lupus Erythematosus (SLE) is a multifaceted autoimmune disorder characterized by widespread inflammation and immune dysregulation, impacting various organ systems and generating autoantibodies. Oral lesions are a common and distressing manifestation of SLE, significantly affecting patients’ quality of life. Cytokines, key mediators of immune responses, play a crucial role in the pathogenesis of both systemic and oral manifestations of SLE. This review sheds the light on current research on the involvement of various cytokines, including interleukins different interferon types, and growth factors in SLE. The intricate interplay between pro-inflammatory and anti-inflammatory cytokines contributes to the disease’s initiation, progression, and diverse clinical presentations. Elevated levels of pro-inflammatory cytokines exacerbate inflammation, promote apoptosis, and drive autoantibody production. Understanding the specific roles of these cytokines offers potential therapeutic targets for managing SLE and improving patient outcomes.

## 1 Introduction

Systemic Lupus Erythematosus (SLE) is a complex autoimmune disease that affects multiple organ systems, including the skin, kidneys, and joints. The systemic nature of SLE arises from widespread immune system dysfunction, leading to autoimmunity against nucleic acids and their associated proteins, along with tissue-damaging inflammation. Among its numerous manifestations, oral lesions are a significant concern for patients, often resulting in pain, discomfort, and diminished quality of life. The pathogenesis of these oral manifestations is multifactorial, with cytokines playing a pivotal role. Recent literature has increasingly highlighted the importance of cytokines in the development and progression of oral lesions in SLE (1). This review aims to synthesize current findings on the role of cytokines in SLE’s systemic and oral manifestations and explore their therapeutic implications. Although genetic susceptibility and environmental factors, such as microbial infections, create a predisposition for SLE, specific triggers of immune activation are necessary to initiate the production of type 1 interferons and the generation of autoantibodies targeting self-antigens (2).

In SLE patients, elevated levels of various cytokines were detected throughout the disease course, appearing in circulation, saliva, urine, and affected tissues such as the skin, kidneys, and synovia (3). While most of these cytokines exhibit pro-inflammatory properties, some play immunomodulatory or anti-inflammatory roles (4).

**Figure 1:**
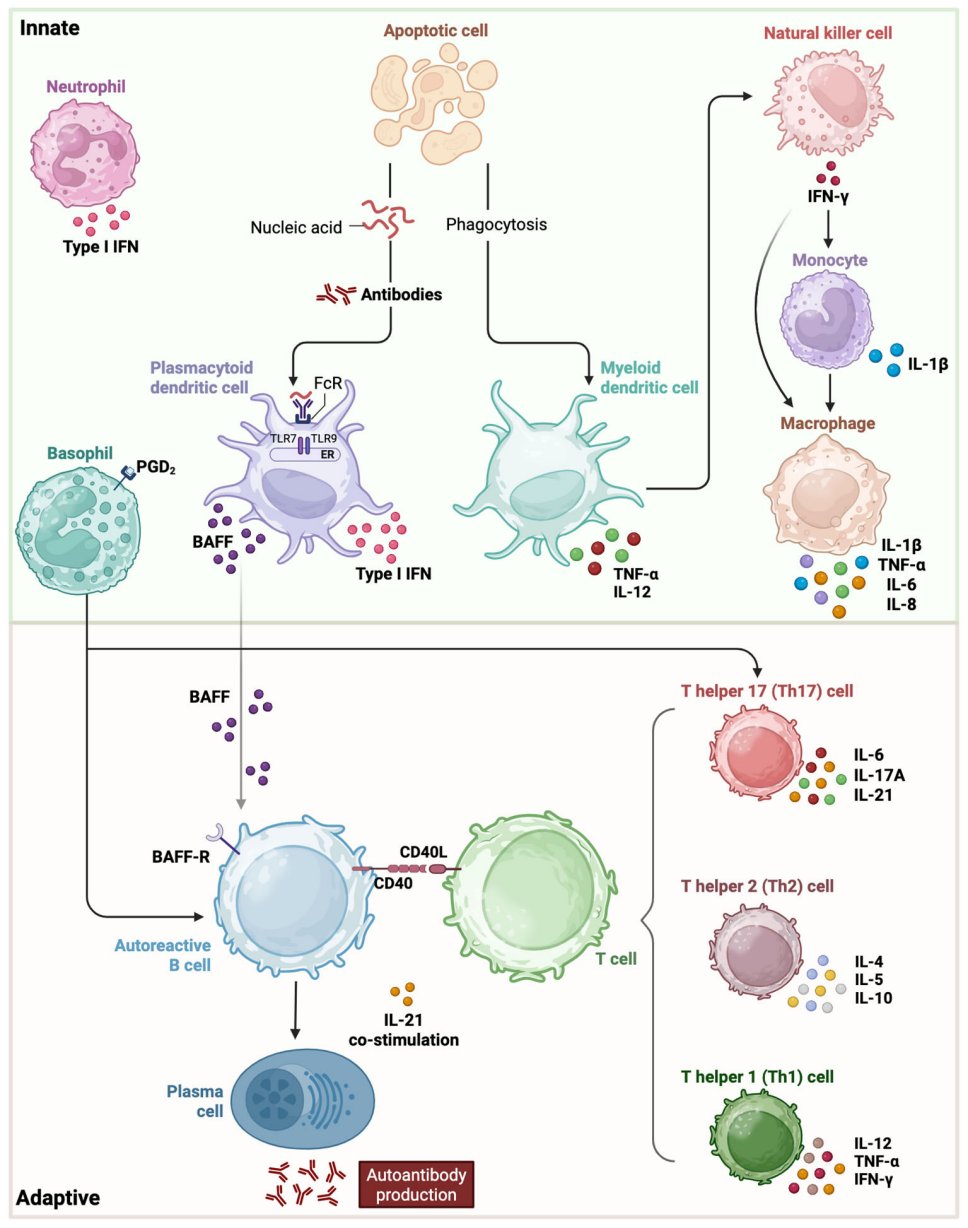
Innate and adaptive immune players with their respective cytokine involvement in SLE pathogenesis. In the innate phase, type I interferons, cell apoptosis and other early signaling pathways initiate activation of basophils, neutrophils, monocytes, macrophages, myeloid and plasmacytoid dendritic cells. The adaptive phase involves an imbalance among T helper cell subsets, their interaction with B cells, perpetuating autoantibody production and tissue damage. BAFF, B-cell-activating factor of the tumor-necrosis-factor family; IFN, interferon; IL, interleukin; PGD_2_, prostaglandin D2; TNF, tumor necrosis factor.

Notably, multiple immunological abnormalities play a role in the breakdown of self-tolerance and the persistence of autoimmune responses in SLE. In particular, defects in apoptotic cell clearance—such as impaired phagocytosis and complement activation—lead to the accumulation of self-antigens. Dysregulated innate immune responses further drive adaptive immune activation, intensifying the inflammatory cascade (5). For instance, plasmacytoid dendritic cells (pDCs), myeloid dendritic cells (mDCs), monocytes and macrophages are key contributors to the inflammatory cascade in SLE through their enhanced antigen-presenting capacity and increased production of proinflammatory cytokines such as type I interferons and TNF-α. These cells exhibit an activated phenotype in SLE patients, promoting T cell activation and perpetuating autoimmunity (6–10). Additionally, abnormalities in B cell development, activation, and differentiation promote the production of autoreactive antibodies, which target self-antigens and form immune complexes that contribute to tissue damage and inflammation. Consistently, disturbances in T cell compartments, dysfunctional regulatory T cells (Tregs), and altered cytokine production further exacerbate tissue damage in this disease. B cells are central to SLE pathogenesis as the overexpression of B lymphocyte stimulator (BLyS) significantly contributes to disease progression (**Figure 1**). BLyS enhances B-cell survival by inhibiting apoptosis and promoting proliferation and differentiation. This process ultimately leads to increased autoantibody production, a hallmark of SLE (11).

## 2 Cytokines involvement in the pathophysiology of lupus erythematosus

SLE is characterized by an aberrant immune system that generates autoantibodies and induces widespread inflammation, affecting various tissues. As shown in **Figure 1**, this immune dysregulation involves the activation of both innate and adaptive immune systems including B and T cells, leading to the formation of immune complexes, and the production of pro-inflammatory cytokines (12, 13). Excessive or insufficient cytokine production can contribute to the pathogenesis of several diseases (14). An imbalance between pro-inflammatory and anti-inflammatory cytokines is a well-recognized feature of SLE. Numerous studies suggest that cytokine levels play a role in SLE, with an elevation of pro-inflammatory cytokines, potentially exacerbating inflammation, promoting apoptosis, and driving autoantibody production, thus contributing to disease initiation and progression (15, 16). These cytokines contribute to the diverse clinical manifestations of lupus, including oral lesions and systemic features. Interleukins, including IL-1, IL-6, IL-10, IL-17, along with interferon-alpha (IFN-α) and tumor necrosis factor-alpha (TNF-α), are key cytokines that serve as biomarkers for assessing disease activity and severity. Therefore, modulating these cytokines could be a therapeutic approach towards the management of SLE (17, 18).

### 2.1 Interferons

#### 2.1.1 Type I interferons

Type I IFNs, particularly IFN-α, are the predominant cytokines of this category and have been extensively studied in the context of SLE (19, 20). Also, IFN-*κ* signaling via the type I interferon receptor modulates the expression and release of cytokines from monocytes (21, 22). All type I IFNs transmit signals through the same receptor complex, composed of IFNAR1 and IFNAR2 (23). Elevated IFN activity has been associated with greater disease severity in SLE patients (24–26). Circulating IFN-α levels have been shown to correlate with disease activity, as measured by the SLEDAI score, as well as with anti-dsDNA antibody titers and complement activity. This suggests that IFN-α quantification could serve as a potential biomarker for disease monitoring (4). Specific autoantibody profiles, including anti-dsDNA, anti-RNP, anti-Sm, and anti-Ro, have been linked to heightened IFN activity (26–28). Multiple studies have demonstrated that between 50% and 75% of adults and up to 90% of children with SLE exhibit heightened expression of type I IFN-regulated genes, a phenomenon known as the IFN signature (29–32). Notably, younger patients display more pronounced IFN activity compared to older individuals, and SLE disease activity has been found to correlate with IFN-α levels and the intensity of the IFN signature (30, 33–35).

In patients with SLE, pDCs are reduced in circulation but can be identified in inflamed tissues such as the skin and kidneys, where they appear to be activated (36–38). These cells play a central role in sustaining IFN production in SLE. Hence, targeting pDCs in SLE patients was found to reduce the expression of IFN response genes in the blood, decrease immune cell infiltration in the skin, and ameliorate cutaneous lesions (39). This approach was done using the monoclonal antibody targeting blood DC antigen 2 (BDCA2), BIIB059, a known pDC-specific receptor that inhibits the production of type I IFNs (39). However, recent research indicated that BDCA2 is not exclusive to pDCs and is also expressed on differentiated monocytes. Consequently, BIIB059 may exert effects on broader myeloid populations, which could have an impact on the safety and expanded therapeutic applications in SLE (40, 41).

On the other hand, other studies demonstrated that pDCs in both preclinical autoimmunity and established SLE are functionally impaired, showing signs of stress, senescence, and reduced cytokine and T cell activation capacity. Instead, non-hematopoietic cells, particularly keratinocytes producing interferon-κ, could drive early interferon responses in the skin, implicating them as key sources of IFN prior to clinical disease onset (42). Also, another study on discoid lupus and cutaneous lupus erythematosus (CLE) identified that pDCs are not major producers of type I interferons, as they exhibit markedly reduced IFN-α production upon toll like receptor 7 (TLR7) stimulation (43).

Neutrophils also possess the capacity to produce type I IFN, and bone marrow-derived neutrophils from SLE patients have been shown to generate IFN-α (44, 45). This IFN production appears to drive alterations in B-cell development, leading to a reduction in pro/pre-B cells while expanding transitional B-cell populations. Such changes may represent early events in the disruption of immune tolerance and the initiation of autoimmunity, culminating in autoantibody production. A study by Klein B et al. identified Z-DNA binding protein 1 (ZBP1) as a key mediator of UVB-induced inflammation in autoimmune photosensitivity by stabilizing cytosolic Z-DNA derived from oxidized mitochondrial DNA. Moreover, ZBP1 was found to be elevated in lupus keratinocytes, where it enhances type I IFN production via cGAS-STING signaling, highlighting its central role in priming cutaneous inflammation (46).

Besides, IFN-*κ* is constitutively and highly expressed in keratinocytes (21). In patients with SLE, keratinocytes contribute to skin injury pathogenesis by undergoing apoptosis or necrosis, leading to the release of autoantigens (47). IFN-κ was previously investigated in SLE where it was found to be elevated in the skin lesions of cutaneous lupus erythematosus (48). Further, IFN-κ was found to amplify and accelerate responsiveness of epithelia to IFN-α and increase keratinocyte sensitivity to UV irradiation (48). This indicated that IFN-κ could be a potential novel target for ultraviolet B prophylaxis and cutaneous lupus erythematosus-directed therapy (48). The IFN score was identified to be markedly enriched in the skin of SLE patients, even in the absence of clinical inflammation or in ANA positive individuals (42). Furthermore, IFN-κ was diffusely expressed not only in the epidermis of lesional skin from SLE patients, but also in non-lesional epidermis of ANA+ individuals with elevated systemic IFN activity (42). On another note, IFN-κ functions as an interferon-stimulated gene (ISG) that is regulated in an IFN-β–dependent manner. While IFN-β is produced rapidly and transiently in response to stimulation, IFN-κ expression is upregulated later and sustained, suggesting a potential role in maintaining more prolonged or chronic type I IFN responses (49). This could be the explanation to the elevated levels of IFN-κ in autoimmune diseases such as SLE. Furthermore, the study by Xu B. et al. suggests a mechanistic model in keratinocytes whereby IRF3-activating stimuli rapidly induce IFN-β leading to the subsequent upregulation of IFN-κ via IFN-β–mediated STAT1 activation. Further investigation into these pathways may uncover novel therapeutic targets for chronic IFN-driven inflammation in cutaneous lupus (49).

#### 2.1.2 Type II interferons

In contrast, type II IFN consists of a single cytokine, IFN-γ, which is primarily secreted by CD4^+^ and CD8^+^ Th1 lymphocytes, as well as by NK cells, B cells, and professional antigen-presenting cells. IFN-γ signaling occurs through a receptor complex consisting of IFNGR1 and IFNGR2 subunits (20). Activated NK cells in SLE exhibit increased secretion of IFN-γ, while peripheral blood leukocytes from SLE patients have been found to express detectable levels of IFN-λ transcripts, although the precise cellular source remains uncertain (50). A crucial observation is that several activated immune cell types can further stimulate pDCs to enhance IFN production. Notably, NK cells, B cells, and T cells have all been shown to amplify IFN secretion when pDCs are exposed to nucleic acid–containing immune complexes (51, 52). Interleukin-12, alongside IFN-γ, drives T-cell differentiation toward a Th1 immune response. SLE patients exhibited an increase in the circulating levels of the IL-12p40 subunit, which correlates positively with disease activity and inversely with complement C3 levels (53, 54).

#### 2.1.3 Type III interferons

In established SLE, there is a production of type III IFNs (IFN-λ) in the skin. This type of IFNs engages signaling pathways and elicit cellular effects similar to type I interferons (55). Notably, studies using the SLE animal model (MRL/lpr), reported that deletion of the IFN-λ receptor leads to reduced skin inflammation, highlighting its functional relevance (56). Additionally, loss of *IFNB1* (gene for IFN-β1) in keratinocytes significantly diminished *IFNL1* (gene for IFN-Λ1) expression, mirroring the pattern seen with IFN-κ. In contrast, *IFNL3* remained strongly and rapidly induced even in the absence of *IFNB*. These findings underscore the complex interplay between type I and type III interferons in the epidermis, warranting further investigation.

### 2.2 Growth factors

Increasing evidence suggests that TNF-α plays a complex role in the pathogenesis of SLE (57). This cytokine appears to have dual and sometimes opposing functions, making its role in the disease a subject of ongoing debate. On one hand, TNF-α contributes to immune regulation by supporting the development, differentiation, and maintenance of immune cells, acting as a crucial component of immunosuppressive mechanisms. On the other hand, it can serve as a potent proinflammatory agent, being released in affected tissues during active disease and potentially exacerbating inflammation (58–60).

Also, studies have shown that individuals with active SLE tend to exhibit elevated serum levels of TNF-α and its soluble receptors (TNFR1 and THFR2) compared to those with inactive disease (57, 61–63). A study by Zhu et al. revealed that SLE patients exhibited significantly reduced expression of key TNF adaptor proteins including TNF receptor-associated death domain (TRADD), Fas-associated death domain (FADD), TRAF2, and receptor-interacting protein kinase 1 (RIPK1) in their peripheral blood mononuclear cells. Furthermore, these diminished protein levels were found to correlate inversely with disease activity, suggesting a potential link between impaired TNF signaling and SLE progression (64). However, conflicting findings suggest that TNF-α levels may be lower in certain SLE patients, particularly those experiencing severe disease manifestations. Interestingly, some research indicates that TNF-α concentrations are higher in individuals with inactive SLE than in both those with highly active disease and healthy controls (57, 58, 65). This paradoxical pattern has led to speculation that TNF-α may also play a protective role in certain contexts, potentially contributing to immune regulation in SLE.

Both the transmembrane and soluble form of TNF interact with the two known receptors of TNF, TNF receptor 1 (TNFR1), and TNFR2 (66). They play distinct yet complementary roles in immune regulation and inflammation (67). TNFR1 (p55), is ubiquitously expressed on most cell types and mediates the majority of pro-inflammatory and apoptotic effects of TNF-α through activation of NF-κB, MAPK, and caspase pathways. In contrast, TNFR2 (p75) is primarily expressed on immune cells, including Tregs, endothelial cells, and certain neuronal populations, and is associated with tissue regeneration, immune modulation, and cell survival via alternative NF-κB and PI3K/Akt signaling pathways (68, 69). Overactivation of TNFR1 contributes to heightened inflammation, apoptosis of key immune and tissue cells, and amplification of the autoimmune response. Meanwhile, dysregulation or insufficient activation of TNFR2 may impair Treg function and tissue repair mechanisms, exacerbating disease progression in SLE (70). Emerging studies suggest that selective modulation of TNFR2 could restore immune tolerance without triggering widespread inflammation, offering a promising therapeutic strategy for SLE and other autoimmune diseases (71).

CD40, a transmembrane receptor of the TNF family, is broadly expressed on immune and non-immune cells and plays a pivotal role in regulating both humoral and cellular immunity through its interaction with CD40 ligand (CD40L) (72–74). This pathway is essential for dendritic cell/T-cell and T-cell/B-cell communication, promoting B cell proliferation, differentiation, and immunoglobulin production (73, 75). In SLE, CD40 signaling contributes to disease pathology by as its activation upregulates CD40L, which in turn further engages CD40 on B cells (76). Additionally, CD40-induced telomerase activity supports long-lived B cell memory, perpetuating chronic inflammation and autoantibody generation (77).

### 2.3 Interleukins

#### 2.3.1 Interleukins belonging to IL-1 family

The cytokine interleukin-1 superfamily comprises IL-1α, IL-1β, IL-18, IL-33, and IL-38, all of which contribute to innate immunity and are regulated by inflammasome activation as an early pathogen response. Some studies reported that peripheral IL-1α levels in SLE patients were comparable to those of controls, while other studies indicated elevated IL-1α levels in individuals with renal and joint manifestations in SLE (78). Additionally, increased tissue IL-1β levels have been observed in skin lesions triggered by photoprovocation in CLE, accompanied by heightened TNF-α expression (79). In SLE, opsonized red blood cells retaining mitochondria (Mito^+^ RBCs) stimulate monocytes to co-produce type I interferons and mature IL-1β. This response is driven by cyclic GMP-AMP synthase (cGAS) and RIG-I-like receptors detecting mitochondrial DNA and RNA from Mito^+^ RBCs, activating both interferons signaling and inflammasome pathways. Notably, IL-1β secretion occurs through the activation of an IFN-inducible myxovirus-resistant protein 1 (MxA) and trans-Golgi network dependent mechanisms that are independent of gasdermin D or pyroptosis. This sheds the light on a subset of monocytes that express IFN-stimulated genes (ISGs) and released IL-1β and are enriched in patients with active SLE (8).

IL-18 is secreted by macrophages and plays a crucial role in promoting IFN-γ production by Th1 cells and splenocytes, often acting synergistically with IL-12 (80). Elevated serum IL-18 levels are commonly observed in SLE patients, particularly in those with active renal disease who exhibit an increased risk of developing kidney damage over time (81). Additionally, high IL-18 expression has been detected in cutaneous lupus erythematosus (CLE) lesions (82). IL-18 is identified as a potential biomarker for active SLE, whereas blocking IL-18 was found to delay the onset of SLE-like autoimmunity (80, 83).

#### 2.3.2 Interleukins belonging to Type I cytokine family

Elevated plasma IL-6 levels have been detected in patients with renal impairment and are a key mediator in lupus nephritis (78, 84, 85). Urinary IL-6 levels have been proposed as a potential biomarker for the condition (86, 87). Additionally, SLE has been linked to impaired IL-2 production, with IL-2 deficiency associated with renal dysfunction (88). Notably, some SLE patients develop anti-IL-2 autoantibodies, which have been correlated with disease activity (89). IL-21 is an autocrine cytokine primarily produced by follicular helper T (Tfh) cells and Th17 cells, playing a key role in their development. It also facilitates B-cell differentiation into plasma cells through various signaling pathways, including JAK/STAT (90). IL-21 may contribute to the expansion of autoreactive B cells, further driving disease pathology (91). Genetic variations in IL-21 and its receptor (IL-21R) have been linked to increased susceptibility to SLE. There are controversial reports regarding the expression of IL-21 where some studies found elevated levels of IL-21-producing cells in the circulation of SLE patients (92), while others have found reduced circulating IL-21 levels in affected individuals (93).

#### 2.3.3 Interleukins belonging to Type II cytokine family

Despite its anti-inflammatory functions, IL-10 can have pro-inflammatory effects, potentially influenced by type I IFNs (94). The genetic variations in the IL-10 gene are linked to increased SLE susceptibility (95, 96). Plasma IL-10 concentrations were elevated in SLE patients compared to healthy controls, and there was an association between IL-10 levels, disease activity, and anti-dsDNA antibody titers (78).

#### 2.3.4 Interleukins belonging to IL-17 family

Another proinflammatory cytokine is IL-17 which plays a key role in the pathogenesis of autoimmune rheumatic diseases, including SLE (18). Its involvement in SLE development and disease activity is supported by findings that SLE patients exhibit significantly higher circulating IL-17 levels compared to healthy individuals, with its levels correlating positively with disease severity (97). Beyond driving inflammation, IL-17 also contributes to the progression of SLE-related comorbidities (98). The differentiation of naïve T cells into Th17 cells is driven by the combined effects of TGF-β and IL-6, while IL-23 is essential for the maturation and activation of pathogenic Th17 cells. IL-6 plays a crucial role in the differentiation of Th17 cells, and its inhibition may help mitigate SLE activity by indirectly suppressing IL-17-driven inflammation (99). IL-17, either alone or in conjunction with B-cell activating factor (BAFF), regulates B-cell survival, proliferation, and differentiation into immunoglobulin-secreting cells (97). By promoting B-cell expansion, IL-17 enhances autoantibody synthesis, which in turn facilitates immune complex formation, complement activation, and subsequent tissue damage in target organs. Additionally, these immune complexes activate plasmacytoid dendritic cells (pDCs), further fueling inflammation (100). The IL-23/IL-17 axis plays a central role in inflammation, coordinating Th17 cell differentiation and function, thereby perpetuating immune dysregulation in SLE (11). IL-17 interacts with other cytokines such as IL-23, IL-17F, and IL-21 to form a complex proinflammatory network that drives tissue damage and inflammation in SLE. It also amplifies the inflammatory cascade by inducing the release of other cytokines, such as IL-1 and IL-6. IL-21 plays a crucial role in maintaining the balance between Th17 and Treg cells, while IL-10, a member of the IL-2 cytokine family, is linked to chronic inflammation (97, 100).

Higher plasma IL-17 levels and an increased presence of circulating Th17 cells were reported in SLE patients (101–104). Multiple investigations have shown that serum IL-17A levels are markedly higher in patients with active SLE than in healthy individuals (105–107). Furthermore, IL-17 concentrations have been positively correlated with SLE disease activity index (SLEDAI) scores, indicating a direct link between IL-17 expression and disease severity (101, 108, 109). In newly diagnosed SLE patients, IL-17A levels were also found to be strongly associated with RORγt mRNA expression, erythrocyte sedimentation rate, and immunoglobulin levels (IgG and IgA), further emphasizing the crucial role of the Th17–IL-17 axis in SLE pathogenesis (110).

## 3 Oral manifestations of lupus erythematosus

Oral manifestations of SLE are common and range from non-specific findings such as xerostomia (dry mouth) and ulcers to more specific lesions like oral lichen planus and candidiasis (111). Oral manifestations of SLE can provide valuable clues for diagnosis and management. These manifestations can include oral ulcers, lupus cheilitis, discoid lupus erythematosus (DLE), dry mouth (xerostomia), gingival inflammation, periodontal disease, pale mucosa, oral candidiasis, telangiectasia (dilated blood vessels), and altered taste (dysgeusia) (112).

Aphthous-like ulcers are painful, round, or oval lesions often found on the hard palate, buccal mucosa, and tongue. These are similar to typical aphthous ulcers but can occur in a variety of sizes. Painless or painful ulcerations may appear as shallow, necrotic, and have a raised border (112).

Lupus cheilitis is characterized by red, scaly, or fissured lips. The lower lip is often more affected. This may resemble a form of cheilitis, with dry, cracked, and inflamed lips. DLE may present as localized lesions on the face, scalp, or mucous membranes, including the oral cavity. White, lacy lesions can be seen on the buccal mucosa and palate, and they may be accompanied by erythema or ulceration. These lesions are often referred to as lichen planus-like lesions.

Due to autoimmune damage to salivary glands, SLE can result in decreased saliva production, leading to a sensation of dryness in the mouth, difficulty swallowing, and increased risk of dental caries and oral infections. Gingivitis may be present, characterized by redness, swelling, and bleeding of the gums, often exacerbated by the systemic inflammation associated with SLE. Moreover, SLE patients may be at an increased risk for periodontal disease due to both autoimmune factors and dry mouth (xerostomia), which can lead to a higher incidence of plaque accumulation and bacterial growth. In addition, immunosuppression due to SLE or its treatment (such as corticosteroids) can increase the risk of opportunistic infections like oral thrush (candidiasis). Occasionally, visible dilated blood vessels may appear on the hard and soft palate. Some patients may experience changes in their sense of taste, which can be related to the disease or its treatment. Further, mucosal tissues can appear pale due to anemia, a common complication of SLE, reflecting the decreased red blood cell count.

### 3.1 Role of cytokines in oral manifestations of SLE

The relationship between cytokines and oral lesions in lupus suggests that these cytokines may not only reflect disease activity but also contribute to the inflammation and tissue damage observed in the oral mucosa (113).

Type I interferons, particularly IFN-α and IFN-β, play a crucial role in the immunopathogenesis of lupus and its related oral lesions. These cytokines enhance antigen presentation and promote the activation of autoreactive immune cells, contributing to ongoing inflammation. Elevated levels of IFN-α have been observed in lupus patients with oral manifestations, correlating with increased mucosal damage and immune dysregulation (114). Additionally, type I interferons stimulate the production of other inflammatory mediators, reinforcing the chronic immune activation observed in the oral mucosa (115).

TNF-α is a significant cytokine involved in lupus oral pathology. It promotes the apoptosis of keratinocytes, resulting in ulcer formation and destruction of mucosal tissue. Studies have shown significantly elevated levels of TNF-α in saliva and serum samples from lupus patients with oral lesions, indicating its role in amplifying inflammatory responses in the oral mucosa (116). Targeting TNF-α with biologic therapies has been investigated as a potential strategy for alleviating both oral and systemic lupus symptoms (117).

IL-6 is a critical cytokine involved in the chronic inflammation associated with lupus oral lesions. It drives B-cell hyperactivity and promotes autoantibody production, exacerbating immune-mediated tissue damage. Salivary and tissue samples from lupus patients show elevated IL-6 levels, further highlighting its contribution to disease progression (118). This cytokine also interacts with type I interferons, creating an inflammatory loop that sustains mucosal lesions (119). IL-8, a potent chemoattractant for neutrophils, plays a crucial role in the chronic inflammatory process associated with lupus oral lesions. Its overexpression in oral mucosal tissues has been linked to excessive infiltration of immune cells and prolonged persistence of lesions (117). IL-8 recruits neutrophils and monocytes to the affected tissues, exacerbating tissue damage and further sustaining the inflammatory environment (120).

IL-1 is a key mediator of inflammation and has been implicated in the pathogenesis of oral lesions in lupus. It exists in two primary forms, IL-1α and IL-1β, both of which enhance immune cell activation and amplify the release of inflammatory cytokines. IL-1 has been shown to potentiate the effects of TNF-α and IL-6, worsening mucosal tissue damage in lupus patients (121). The elevated levels of IL-1 in saliva and oral biopsies support its role in promoting ulceration and chronic inflammation (122). IL-18 is recognized as a crucial cytokine that influences the severity of oral lesions in lupus. IL-18 promotes the production of IFN-γ, leading to increased immune activation and progressive tissue destruction (123). Elevated IL-18 levels in lupus patients correlate with the severity of oral lesions, underscoring its potential as a biomarker for disease monitoring (124).

IFN-γ serves as a key regulator of Th1-mediated immune responses in lupus. It promotes macrophage activation and elevates the expression of adhesion molecules that facilitate immune cell infiltration into oral tissues. The increased levels of IFN-γ in lupus oral lesions indicate its role in sustaining chronic inflammation and immune dysfunction (125). Additionally, IFN-γ works in synergy with type I interferons, establishing a self-sustaining inflammatory cascade that contributes to the persistence of oral lesions (126). Furthermore, IL-17 contributes to the recruitment of neutrophils and macrophages, sustaining chronic inflammation and tissue destruction in the oral mucosa (127). Elevated levels of IL-17 in lupus patients have been linked to disease severity and increased lesion persistence, making it an emerging therapeutic target (128).

Macrophage inflammatory protein-1 alpha (MIP-1α), also known as CCL3, plays a significant role in the recruitment of immune cells and the inflammation associated with lupus oral lesions. Its elevated expression correlates with increased leukocyte infiltration and persistent mucosal inflammation (117). By promoting the migration of immune cells to inflamed oral tissues, MIP-1α aids in the maintenance of chronic immune activation (129). These cytokines work together to contribute to the complex immunopathology of oral lupus lesions by sustaining chronic inflammation, disrupting mucosal homeostasis, and driving disease progression. Understanding their roles offers insight into potential therapeutic strategies to alleviate oral manifestations in lupus patients.

## 4 Systemic manifestations of lupus erythematosus

SLE is characterized by widespread organ involvement, primarily driven by cytokine dysregulation. Key cytokines, including TNF-α, IL-6, IL-17, and type I interferons (particularly IFN-α), contribute to immune complex deposition, chronic inflammation, and tissue injury across multiple systems. In musculoskeletal tissues, these cytokines promote synovitis, joint inflammation, and cartilage degradation (130, 131). Similarly, renal involvement is mediated by glomerular immune complex deposition and cytokine-driven mesangial proliferation, ultimately contributing to lupus nephritis (132, 133). In parallel, within the skin, cytokines induce keratinocyte apoptosis and sustain cutaneous inflammation (134–136). Moreover, cardiovascular complications arise from cytokine-mediated endothelial dysfunction, which promotes the development of atherosclerosis and thrombosis (137, 138). Likewise, pulmonary manifestations reflect cytokine-induced interstitial inflammation, fibrosis, and vascular remodeling (139, 140). In addition, gastrointestinal features include mesenteric vasculitis and mucosal inflammation (141, 142), whereas hematologic abnormalities result from bone marrow suppression and an increased thrombotic risk (143, 144). Finally, Neuropsychiatric symptoms are associated with blood-brain barrier disruption, neuroinflammation, and cerebrovascular damage, which are linked to elevated cytokine levels (145, 146). Altogether, understanding these cytokine-mediated mechanisms provides the basis for targeted therapeutic interventions across the diverse systemic manifestations of SLE.

## 5 Therapeutic targeting of cytokines in SLE

The significant role of cytokines in the pathogenesis of SLE’s systemic and oral manifestations opens up potential therapeutic strategies targeting specific cytokines. Their inhibition has been investigated in preclinical models and targeted biologic therapies have been developed and assessed in clinical trials. While current treatments for lupus, such as hydroxychloroquine and immunosuppressants, aim to reduce systemic inflammation, biologic therapies that target cytokines have shown promise in treating both systemic and oral manifestations (3). **Table 1** summarizes the key cytokine-targeted biologics in SLE with their mechanisms and clinical applications. Cytokine-targeted therapies, particularly IL-1 blockers (e.g., anakinra), and TNF-α inhibitors, represent a promising approach for reducing the severity of oral lesions and improving the oral health of lupus patients (147). A major challenge in SLE clinical trials has been the inclusion of heterogeneous patient populations, which may obscure treatment effects. To address this, it has been suggested that patients be stratified based on clinical and genetic phenotypes or cytokine profiles before trial enrollment (24, 148).

**Table 1:** Cytokine-targeted biologics in lupus erythematosus: mechanisms and clinical applications.

Therapy/biologic	Target cytokine/ pathway	Mechanism of action	Clinical application in lupus	Relevance to oral/ mucocutaneous features
Belimumab	BAFF (B-cell activating factor)	Inhibits B-cell survival and differentiation	Approved for systemic lupus; effective in reducing flares	May reduce mucocutaneous activity indirectly
Anifrolumab	Type I Interferon receptor	Blocks type I IFN signaling, a key inflammatory driver	Approved for moderate-to-severe SLE	Shown to improve mucocutaneous manifestations
Tocilizumab	IL-6	Blocks IL-6 receptor, reducing inflammation	Investigational for SLE and lupus nephritis	Potential benefit in refractory oral ulcers
Ustekinumab	IL-12/IL-23	Inhibits Th1 and Th17 pathways	Investigational; used in SLE with cutaneous and joint involvement	May improve oral and skin lesions
Rituximab	CD20 (B cells)	Depletes B cells, reducing autoantibody production	Off-label for refractory SLE	Indirect benefit to oral ulcers via systemic disease control
Anakinra	IL-1	IL-1 receptor antagonist	Limited use in SLE; considered for inflammatory syndromes	Possible role in severe mucosal inflammation
TNF-α inhibitors	TNF-α	Neutralize TNF-α to reduce inflammation	Rarely used due to lupus-like reactions; may benefit arthritis	Not typically used due to paradoxical lupus risk
IFN-γ blocking agents	Interferon-gamma	Reduces Th1-driven inflammation	Investigational stage	Theoretical benefit in mucosal lesions, under study

Guselkumab is a monoclonal antibody that binds with high affinity to human IL-23, preventing its interaction with the cell surface receptor. By blocking IL-23-mediated signaling, guselkumab inhibits the activation and cytokine production associated with this pathway. This is being explored in a phase 2 clinical trial (NCT04376827) by evaluating the safety and efficacy of guselkumab in combination with standard-of-care therapy, compared to a placebo plus standard-of-care (149). Ustekinumab, which targets the p40 subunit shared by IL-12 and IL-23, was evaluated in a phase 2 trial as an add-on therapy to standard SLE treatment. The study demonstrated positive effects on both clinical and laboratory markers, particularly in improving cutaneous and articular manifestations, while maintaining a favorable safety profile. Responders showed a decline in IFN-γ levels (150). Furthermore, ustekinumab suppresses both the Th1 and Th17 pathways (11). Emerging evidence supports the potential of IL-17A as a therapeutic target for SLE, particularly in patients whose disease is primarily driven by IL-17 (99). This was tried directly in the SELUNE study (NCT04181762), a randomized, double-blind trial designed to assess the efficacy and safety of secukinumab (Cosentyx), an anti-IL-17A monoclonal antibody, in combination with standard-of-care therapy for patients with active lupus nephritis (151). Another approach to indirectly target IL-17 involves inhibiting the synthesis of Th17 cells. Specifically, blocking cytokine pathways such as IL-6, IL-1, or IL-23 can interfere with Th17 cell development, thereby reducing IL-17 levels (99).

Tocilizumab, a monoclonal antibody that inhibits IL-6 signaling, was first evaluated for its efficacy in SLE patients in 2010 (152, 153). While it may be beneficial for certain patient subgroups with high inflammatory activity, caution is necessary, as higher doses can lead to immunosuppression (153). Targeting IL-6 with agents such as tocilizumab may help alleviate chronic oral inflammation and promote mucosal healing. Sirukumab is another human monoclonal antibody designed to selectively and effectively target IL-6. By inhibiting STAT-3 phosphorylation, it neutralizes IL-6 activity and mitigates its biological effects (154). To date, no IL-6-targeting therapies have received approval for SLE treatment. On the other hand, clinical trials investigating an anti-IL-10 monoclonal antibody showed a reduction in disease activity among SLE patients. However, the development of anti-drug antibodies raises concerns, and further studies are needed to assess long-term treatment feasibility (155).

The potential of anti-TNF-α monoclonal antibodies to suppress immune mechanisms initially suggested they could be beneficial for SLE treatment. While some studies yielded less promising results, early expectations remained optimistic (59). Studies have demonstrated the effectiveness of anti-TNF-α therapies in treating connective tissue diseases (CTD) (156), particularly SLE and cutaneous lupus erythematosus (CLE) (157). Among the most extensively studied TNF-α inhibitors are infliximab and etanercept (59). Infliximab, due to its chimeric nature, has the highest immunogenicity among anti-TNF-α agents. However, research indicates that infliximab maintains a favorable safety and tolerability profile in SLE patients. Notably, short-term induction therapy with infliximab, in combination with azathioprine or methotrexate, led to sustained improvement in lupus nephritis (57, 158, 159). Moreover, drugs such as etanercept, which target TNF-α, have been shown to reduce oral ulcers and improve overall oral health in lupus patients (160).

Etanercept has also been evaluated in several clinical trials for lupus nephritis treatment (NCT00447265) and discoid lupus erythematosus (DLE) (NCT02656082 and NCT00797784). An observational study found long-term etanercept therapy to be relatively safe and effective for refractory lupus arthritis (57). A separate study investigating SLE patients treated with adalimumab and etanercept revealed a significant reduction in the median prednisone dose, from 15 mg/day to 5 mg/day, during the observation period (59, 161). These findings support the notion that anti-TNF-α therapies may play a role in managing refractory lupus arthritis (59).

Etanercept has also shown efficacy in treating rhupus, a condition that exhibits features of both rheumatoid arthritis (RA) and SLE (162). Additionally, a combination of etanercept, plasmapheresis, and high-dose intravenous gamma globulin has been successfully used to manage severe diffuse proliferative nephritis in pregnant SLE patients (57, 163). Furthermore, a case report highlighted that etanercept could alleviate clinical symptoms and enhance overall quality of life in individuals with subacute cutaneous lupus erythematosus (SCLE) (164).

TNF-α inhibitors have been linked to the development of ANA and anti-dsDNA antibodies, potentially leading to clinical manifestations resembling idiopathic lupus. When this occurs, the condition is referred to as anti-TNF-α-induced lupus (ATIL) (59). This condition, also known as anti-TNF-α-induced lupus erythematosus (ATIL), is typically diagnosed based on a distinct temporal association between symptom onset and the initiation or dosage escalation of anti-TNF-α therapy (57, 157). Picardo et al. conducted a study to evaluate the incidence and clinical-serological features of ATIL in patients undergoing anti-TNF-α treatment. Their findings indicated a higher prevalence of ATIL among patients treated with infliximab compared to those receiving adalimumab (165). Current TNF-α blockers contribute to these side effects by blocking TNF-α interaction with both its regulatory receptor (TNFR2) and its pro-inflammatory/pro-apoptotic receptor (TNFR1) (57).

Rontalizumab and anifrolumab, which block type I interferons, have shown potential in reducing both systemic and oral manifestations of lupus (2).

Clinical trials targeting IFN-γ have begun to yield promising results in SLE treatment. AMG 811, a fully human IgG1 monoclonal antibody against IFN-γ, has exhibited good tolerability in patients with mild to moderate SLE. A single dose of AMG 811 has been shown to normalize IFN-regulated gene expression and induce a dose-dependent decrease in serum CXCL10 levels (166, 167). While AMG 811 effectively modulated IFN-γ-associated biomarkers and maintained a favorable safety profile, it did not produce significant clinical benefits for patients with DLE (168). However, encouraging results from phase Ib trials suggest that inhibiting the IFN-γ pathway may hold therapeutic potential for extrarenal manifestations of lupus (169). Given IFN-γ’s critical role in LN, further exploration of its inhibition in this context is warranted, especially considering the tolerability of its targeted blockade. Two completed studies registered as NCT00818948 and NCT02291588, have evaluated the safety of AMG 811 in SLE treatment.

It is worth mentioning that many other targeted therapies have been used or investigated in SLE treatment. These include targeting CD20, BAFF and cytokine downstream signaling molecules that play a role in the pathogenesis of SLE (149).

## 6 Conclusion

**Figure 2:**
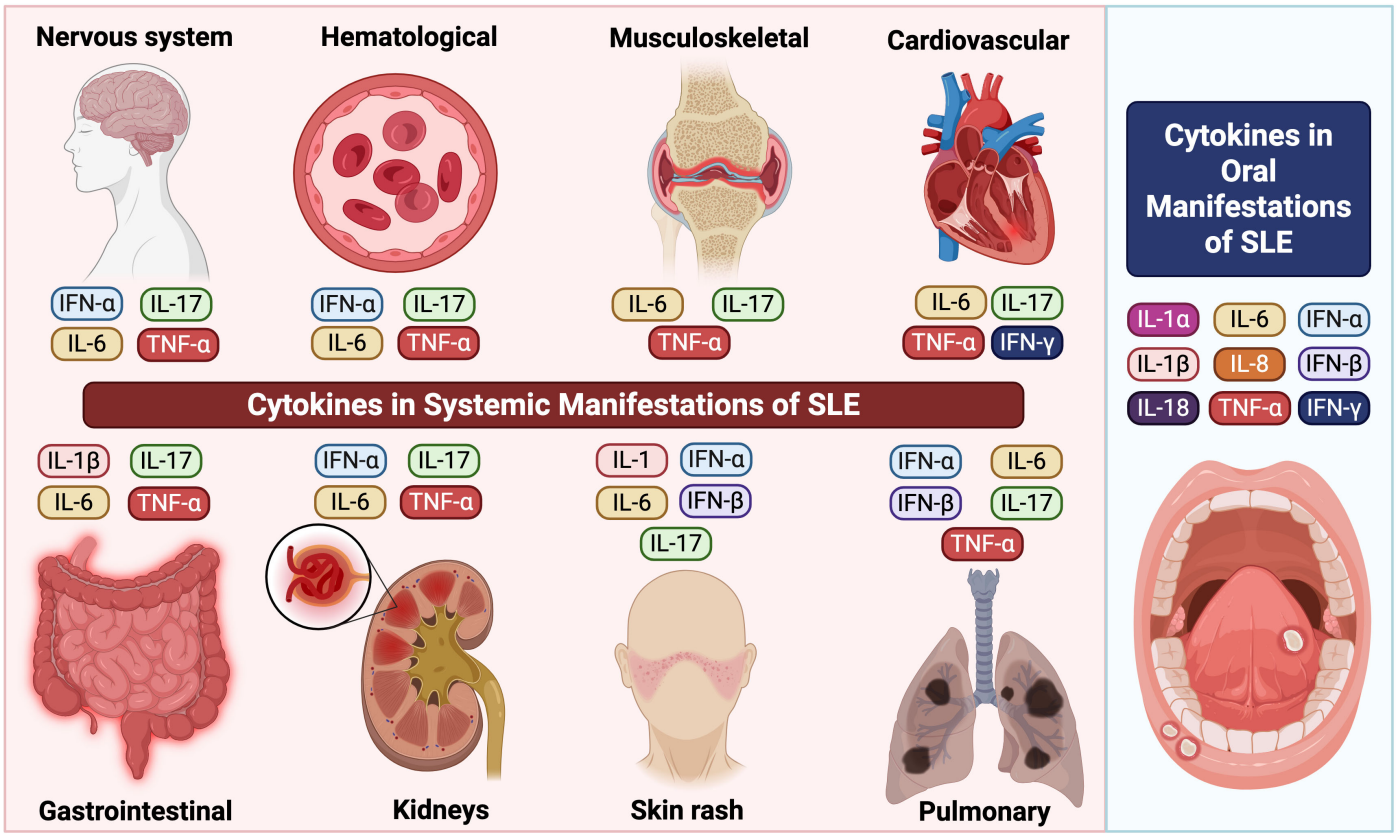
Cytokine-mediated immune mechanisms in SLE manifestations including oral and systemic compartments.

In summary, cytokines exert a profound influence on the pathogenesis of SLE, affecting both systemic and oral manifestations (**Table 2**, **Figure 2**). The imbalance between pro-inflammatory and anti-inflammatory cytokines, particularly the elevation of IFN-α, IL-6, IL-17, and TNF-α, contributes significantly to the disease’s progression and severity. Further research into the precise mechanisms of cytokine involvement is crucial for developing targeted therapeutic strategies. Modulating cytokine activity holds promise for improving the management of SLE, alleviating symptoms, and ultimately enhancing the quality of life for individuals affected by this complex autoimmune disease.

**Table 2:** Organ system manifestations and treatments in lupus erythematosus with immunological focus.

Organ system	Common manifestations	Standard treatments	Immune-modulating therapies (cytokine-targeted)
Oral/Mucocutaneous	Oral ulcers, lichenoid lesions, erythema, cheilitis	Topical corticosteroids, antimalarials	Anti-TNF agents, IL-6 inhibitors (e.g., tocilizumab, in severe cases)
Skin	Malar rash, discoid lesions, photosensitivity	Sunscreens, corticosteroids, antimalarials	Type I IFN blockers (e.g., anifrolumab), Anti-IL-12/23 (ustekinumab)
Musculoskeletal	Arthralgia, arthritis, myositis	NSAIDs, corticosteroids, methotrexate	IL-6 inhibitors, anti-TNF therapies
Renal	Lupus nephritis (proteinuria, hematuria, renal dysfunction)	Immunosuppressants (MMF, cyclophosphamide), corticosteroids	Anti-IFN-α (anifrolumab), BAFF inhibitors (belimumab)
Cardiovascular	Pericarditis, myocarditis, increased atherosclerosis risk	NSAIDs, corticosteroids, statins	Anti-IFN agents, IL-1 inhibitors (e.g., anakinra, in refractory cases)
Pulmonary	Pleuritis, interstitial lung disease	Corticosteroids, immunosuppressants	Anti-IL-6, anti-IFN therapies (investigational)
Hematologic	Anemia, leukopenia, thrombocytopenia	Corticosteroids, IVIG, rituximab	BAFF inhibitors, anti-TNF (in selected cases)
Neuropsychiatric	Seizures, psychosis, cognitive dysfunction	Immunosuppressants, antiepileptics, corticosteroids	Anti-IFN agents, anti-cytokine biologics (case-based)
Gastrointestinal	Abdominal pain, mesenteric vasculitis, lupus hepatitis	Corticosteroids, immunosuppressants	Limited cytokine-targeted therapies; under study
